# Systematic establishment and verification of an epithelial-mesenchymal transition gene signature for predicting prognosis of oral squamous cell carcinoma

**DOI:** 10.3389/fgene.2023.1113137

**Published:** 2023-08-10

**Authors:** Jun Ai, Yaqin Tan, Bo Liu, Yuhong Song, Yanqin Wang, Xin Xia, Qicheng Fu

**Affiliations:** ^1^ Department of Stomatology, Taihe Hospital, Hubei University of Medicine, Shiyan, Hubei, China; ^2^ Department of Urology, Taihe Hospital, Hubei University of Medicine, Shiyan, China; ^3^ Department of Stomatology, Shenzhen Longhua District Central Hospital, Shenzhen, China

**Keywords:** oral squamous cell carcinoma, epithelial-mesenchymal transition, gene signature, prognosis, immune microenvironment

## Abstract

**Objective:** Epithelial-mesenchymal transition (EMT) is linked to an unfavorable prognosis in oral squamous cell carcinoma (OSCC). Here, we aimed to develop an EMT gene signature for OSCC prognosis.

**Methods:** In TCGA dataset, prognosis-related EMT genes with *p* < 0.05 were screened in OSCC. An EMT gene signature was then conducted with LASSO method. The efficacy of this signature in predicting prognosis was externally verified in the GSE41613 dataset. Correlations between this signature and stromal/immune scores and immune cell infiltration were assessed by ESTIMATE and CIBERSORT algorithms. GSEA was applied for exploring significant signaling pathways activated in high- and low-risk phenotypes. Expression of each gene was validated in 40 paired OSCC and normal tissues via RT-qPCR.

**Results:** A prognostic 9-EMT gene signature was constructed in OSCC. High risk score predicted poorer clinical outcomes than low risk score. ROCs confirmed the well performance on predicting 1-, 3- and 5-year survival. Multivariate cox analysis revealed that this signature was independently predictive of OSCC prognosis. The well predictive efficacy was validated in the GSE41613 dataset. Furthermore, this signature was distinctly related to stromal/immune scores and immune cell infiltration in OSCC. Distinct pathways were activated in two subgroups. After validation, AREG, COL5A3, DKK1, GAS1, GPX7 and PLOD2 were distinctly upregulated and SFRP1 was downregulated in OSCC than normal tissues.

**Conclusion:** Our data identified and verified a robust EMT gene signature in OSCC, which provided a novel clinical tool for predicting prognosis and several targets against OSCC therapy.

## Introduction

Oral squamous cell carcinoma (OSCC) represents the predominant type of head and neck squamous cell carcinoma (Park et al., 2019). Surgery, radiotherapy, as well as chemotherapy are the main therapeutic strategies of OSCC (Zhao et al., 2020). The 5-year survival rate is only 50% because of regional invasion as well as lymph node/distant metastases (Liu et al., 2020). Conventional prognostic factors, e.g., stage, are far from optimal (Omori et al., 2020). Although many researches have proposed prognostic markers for OSCC, most of them only focused on several well-studied markers (Almangush et al., 2017). Furthermore, these researches have been carried out in small cohorts, which is difficult to utilize these molecular markers for predicting OSCC prognosis in daily clinical practice (Ju et al., 2020).

Epithelial-mesenchymal transition (EMT) is a dynamic process in which epithelial cells acquire mesenchymal features (Ling et al., 2021), leading to the upregulation of migratory and invasive capacities of tumor cells (Qiao et al., 2020). OSCC primarily contains epithelial dysplasia, loss of epithelial differentiation as well as acquisition of mesenchymal phenotype (Bai et al., 2020). It has been confirmed that EMT process is in relation to OSCC invasiveness and metastasis (Peng et al., 2020). However, it remains vacant concerning the EMT gene signatures and their prognostic value in OSCC. Because of the easy accessibility of gene expression profiles from public databases, exploration of the prognostic gene signatures has been given wide attention (Wu et al., 2019). Based upon the critical role of EMT process in OSCC progression, it is of significance to establish an EMT gene signature for OSCC prognosis. Thus, our research set out to further understand the underlying clinical utility of EMT genes as prognostic markers and to build up individualized clinical outcome evaluation for OSCC.

## Materials and methods

### Data collection

Normalized transcriptome data and clinical information of OSCC were retrieved from the Cancer Genome Atlas (TCGA) database via the UCSC Xena (https://tcga.xenahubs.net) on 11 March 2020. Moreover, the GSE41613 dataset was downloaded from the Gene Expression Omnibus (GEO; http://www.ncbi.nlm.nih.gov/geo/) database (Lohavanichbutr et al., 2013). The specific clinical information was listed in Supplementary Table S1. Before surgery, all patients did not receive radiotherapy or chemotherapy. Primary tumor site of all patients was the same. The “HALLMARK_EPITHELIAL_MESENCHYMAL_TRANSITION” gene set containing 200 genes (Supplementary Table S2) was obtained from the Molecular Signatures Database (https://www.gsea-msigdb.org/gsea/msigdb/index.jsp).

### Data preprocessing

In the TCGA dataset, 328 OSCC patients with complete clinical information were regarded as the discovery set. In the GSE41613 dataset, raw microarray data of 97 patients that possessed complete survival information were pre-corrected, transformed by log2 and normalized, followed by gene annotation. This dataset was utilized as the validation set.

### Establishment of an EMT gene model

Prognosis-related EMT genes were firstly screened in OSCC. Univariate cox analyses were employed to screen EMT genes with *p* < 0.05 in the TCGA dataset. By applying glmnet package, an optimal prognostic model was built with least absolute shrinkage and selection operator (LASSO) Cox regression analysis based on the prognosis-related EMT genes (Friedman et al., 2010). The optimal value of penalty parameter *λ* was determined via ten-fold cross-validation. The risk score of OSCC samples was computed according to regression coefficient as well as expression value of each gene in this model. The formula of the risk score was as follows: risk score = $\sum_{i=1}^{n}\left(LASSO\;coefficient\;of\;gene\;i*expression\;of\;gene\;i\right)$ . OSCC patients in discovery and validation datasets were separated into high- and low-risk subgroups according to the median value of risk score, respectively. High-risk subgroup was defined as patients who had risk scores greater than the median while low-risk subgroup was defined as patients who had risks scores less than the median. To compare the difference in overall survival (OS) time between two subgroups, Kaplan–Meier curves were depicted by survival package and difference was determined with log-rank tests. Time-dependent receiver operating characteristic (ROC) curves of 1-year, 3-year and 5-year OS time were conducted for calculating the area under the curve (AUC) values to assess the predictive efficiency of the gene signature and other clinical features (age, gender, grade and stage) by applying survivalROC package (Heagerty and Zheng, 2005). Furthermore, univariate cox analyses were presented for evaluating the relationships of OSCC prognosis with the gene signature and clinical features. To validate whether the gene signature could be independently predictive of patients’ prognosis, multivariate cox analyses were performed based on prognosis-related factors with *p* < 0.05. Hazard ratio (HR) as well as 95% confidence interval (CI) was computed. Factors with HR > 1 were risk factors and those with HR < 1 were protective factors.

### Independence of the EMT gene signature from other clinical features

For determining whether the gene signature was independent of other clinical features, age, gender, grade, and stage were separated into high- and low-risk subgroups in the discovery dataset. OSCC subjects were stratified into age >65 and <65 subgroups, female and male subgroups, grade I-II/III-IV subgroups, stage I-II/III-IV subgroups. Kaplan-Meier OS analysis was carried out in each subgroup and difference was evaluated by log-rank test.

### Assessment of stromal score, immune score, and tumor purity

Estimation of STromal and Immune cells in MAlignant Tumor tissues using Expression data (ESTIMATE) package (https://sourceforge.net/projects/estimateproject) (Yoshihara et al., 2013) was employed for inferring stromal score, immune score, and tumor purity in each OSCC sample from the TCGA dataset based on gene expression profiles.

### Characterization of immune cell compositions in OSCC tissues

By applying CIBERSORT package (https://cibersort.stanford.edu/) (Newman et al., 2015), the infiltration levels of different immune cells were inferred in OSCC tissue specimens from the TCGA dataset according to gene expression profiling. Only specimens with *p* < 0.05 could be retained for further analyses. LM22 leukocyte gene signature matrix was used as the reference, which contained 547 genes that distinguished 22 human hematopoietic cells as follows: 7 kinds of T cell types, naïve and memory B cells, plasma cells, NK cells as well as myeloid subsets.

### Pathway enrichment analysis

Gene Set Enrichment Analysis (GSEA) software was utilized for exploring the activated signaling pathways through comparing high- and low-risk subgroups from the TCGA dataset (Subramanian et al., 2005). The KEGG gene set (c2. cp.kegg.v7.1. symbols) was used as reference. 1,000 gene-set permutations were carried out. The terms with normalized enrichment score |NES| > 1.5 and FDR <0.05 were chosen as distinct pathways activated in high- or low-risk phenotypes, which were used for multiple GSEA gene sets.

### Somatic mutation analysis

Mutation Annotation Format (MAF) of OSCC samples was downloaded from TCGA database. These specimens were equally separated into high- and low-risk subgroups. The waterfall plots of two subgroups were depicted for illustrating the different mutation events using the Maftools package (Mayakonda et al., 2018).

### Prognostic values of genes in the prognostic gene signature

Univariate cox analyses were applied to evaluate the prognostic value of each gene in the prognosis-related gene signature for OSCC patients from the TCGA dataset. Moreover, their expression was visualized in OSCC and normal tissue specimens. Spearman correlation between genes in this signature was evaluated in OSCC samples. The protein expression of genes from the prognostic gene signature in OSCC tissues was assessed through the Human Protein Atlas (https://www.proteinatlas.org/) (Colwill and Gräslund, 2011).

### OSCC tissue specimens

Totally, 40 paired OSCC and adjacent normal frozen tissues were collected from patients who experienced operation in the Taihe Hospital, Hubei University of Medicine between January 2020 and January 2021. All subjects did not receive chemoradiotherapy before operation. Diagnosis and staging were performed by experienced pathologists according to the American Joint Committee on Cancer Staging System. The study protocol gained the approval of the Ethics Committee of Taihe Hospital, Hubei University of Medicine (KY 2020-024), with written informed consent acquired from each subject. In addition, the research followed the Declaration of Helsinki.

### Real-time quantitative polymerase chain reaction (RT-qPCR)

Each gene in this prognostic model was verified in OSCC by RT-qPCR. Total RNA was isolated from OSCC tissues utilizing TRIzol reagent (Beyotime, China), which was reverse transcribed into cDNA utilizing primers and SuperScriptIII reverse transcriptase. RT-qPCR was carried out through Prime Script RT Reagent Kit and 7500 Real-Time PCR System (Applied Biosystems, United States). GAPDH served as the reference control. These amplification procedures included: denaturation at 95 °C lasting 5 min, 40 cycles of denaturation at 95 °C lasting 15 s, annealing at 55 °C lasting 30 s, and extension at 60 °C lasting 1 min. Table 1 listed the primer sequences of target genes. The relative expression was determined with 2^−ΔΔCT^ method.

**TABLE 1:** Primer sequences for RT-qPCR.

Target genes	Primer sequences
AREG	5′-GTG​GTG​CTG​TCG​CTC​TTG​ATA-3′ (F)
	5′-CCC​CAG​AAA​ATG​GTT​CAC​GCT-3′ (R)
COL5A3	5′-TGA​CCG​GGC​ATT​CAG​AAT​TGG-3′ (F)
	5′-CGG​GCA​CCC​CTT​TCA​TCA​T-3′ (R)
DKK1	5′-CCT​TGA​ACT​CGG​TTC​TCA​ATT​CC-3′ (F)
	5′-CAA​TGG​TCT​GGT​ACT​TAT​TCC​CG-3′ (R)
GAS1	5′-ATG​CCG​CAC​CGT​CAT​TGA​G-3′ (F)
	5′-TCA​TCG​TAG​TAG​TCG​TCC​AGG-3′ (R)
GPX7	5′-CCC​ACC​ACT​TTA​ACG​TGC​TC-3′ (F)
	5′-GGC​AAA​GCT​CTC​AAT​CTC​CTT-3′ (R)
PLOD2	5′-CAT​GGA​CAC​AGG​ATA​ATG​GCT​G-3′ (F)
	5′-AGG​GGT​TGG​TTG​CTC​AAT​AAA​AA-3′ (R)
SFRP1	5′-ACG​TGG​GCT​ACA​AGA​AGA​TGG-3′ (F)
	5′-CAG​CGA​CAC​GGG​TAG​ATG​G-3′ (R)
GAPDH	5′-CTG​GGC​TAC​ACT​GAG​CAC​C-3′ (F)
	5′-AAG​TGG​TCG​TTG​AGG​GCA​ATG-3′ (R)

### Immunohistochemistry

From The Human Protein Atlas (https://www.proteinatlas.org/), immunohistochemistry staining of genes in the EMT gene signature in OSCC and normal oral tissues from 10 OSCC patients. Staining, intensity, quantity and location were also obtained. The used antibodies are as follows: AREG (HPA008720), COL5A3 (HPA048256), GAS1 (HPA066902), PLOD2 (CAB025898) and SFRP1 (CAB008116).

### Statistical analysis

Statistical analysis was carried out by R packages (version 3.5.2) and GraphPad Prism software (version 8.0.1). Comparisons between two subgroups were presented via Student’s t-test or Wilcoxon rank-sum test. Kaplan-Meier survival curves were conducted, and survival difference was analyzed through log-rank test. The predictive efficacy was estimated with ROC curves. Spearman’s test was executed for correlation analysis. *p* < 0.05 was considered significant.

## Results

### Establishment of an EMT gene signature for predicting OSCC prognosis

**FIGURE 1:**
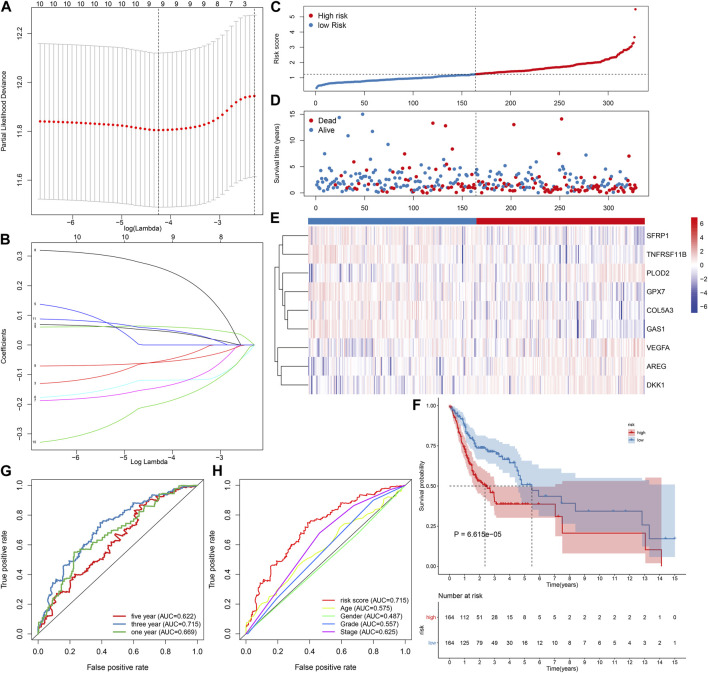
Establishment of an EMT gene signature for predicting OSCC patients’ clinical outcomes in the TCGA-discovery dataset. **(A)** Distribution of partial likelihood deviances corresponding to lambda values. **(B)** Determination of regression coefficients of genes in the LASSO model. **(C)** Distribution of risk scores and determination of high-/low-risk subgroups. Vertical dotted line indicates the median value of risk score. **(D)** Distribution of survival status of high- and low-risk patients. Red dot indicates dead while blue dot is indicative of alive. **(E)** Heat map for expression pattern of these genes in this model in high- (red) and low-risk (blue) subgroups. Red indicates upregulation and blue indicates downregulation. **(F)** OS analysis for high-/low-risk patients. The difference between subgroups was compared with log-rank test. **(G)** ROCs under 1-year, 3-year and 5-year OS for the risk score. **(H)** Comparison of the AUCs among the risk score and other clinical features.

To screen prognosis-related EMT genes in OSCC, univariate cox regression analysis was employed in the TCGA dataset. As a result, 11 genes including ANPEP, AREG, COL5A3, DKK1, FMOD, GAS1, GPX7, PLOD2, SFRP1, TNFRSF11B, VEGFA were significantly associated with OSCC patients’ prognosis (Table 2). These prognostic genes were further assessed by LASSO analysis. Totally, 9 genes were screened for establishing the LASSO model (Figure 1A). The regression coefficient of each gene was calculated, as shown in Figure 1B. The risk score of each patient was then determined, as follows: AREG expression * 0.0471791751815675 + COL5A3 expression * (−0.0543433112313582) + DKK1 expression * 0.0619741505236927 + GAS1 expression * (−0.118881742831249) + GPX7 expression * (−0.142215922759831) + PLOD2 expression * 0.259497264609766 + SFRP1 expression * (−0.0579599175758466) + TNFRSF11B expression * (−0.197512366515929) + VEGFA expression * (0.053308700735239). We separated all patients in the discovery dataset into two subgroups according to the median value of risk score (Figure 1C). Survival status was further compared in the two subgroups. There were more patients with dead status for high-risk in comparison to low-risk subgroups (Figure 1D). Heat map depicted the different expressions of the genes (SFRP1, TNFRSF11B, PLOD2, GPX7, COL5A3, GAS1, VEGFA, AREG and DKK1) in this prognostic model between high- and low-risk subgroups (Figure 1E). Our further analysis demonstrated that high-risk patients exhibited worse survival time in comparison to low-risk subjects (*p* = 6.615e-05; Figure 1F). These data indicated that the risk score could be employed for predicting OSCC patients’ clinical outcomes. We further assessed the predictive efficacy of the risk score for OSCC prognosis by ROCs. The AUCs under 1-year, 3-year and 5-year OS were separately 0.669, 0.715 and 0.622, confirming the well predictive performance for clinical outcomes (Figure 1G). Also, we compared the predictive value of the risk score with other clinical features. Our data demonstrated that the risk score displayed the highest AUC value (0.715) for OS time among age (AUC = 0.575), gender (AUC = 0.487), grade (AUC = 0.557) and stage (AUC = 0.625; Figure 1H), indicating that this signature was more advantageous in comparison to other clinical features regarding prediction of survival time.

**TABLE 2:** Prognosis-related EMT genes in OSCC by univariate cox regression analysis.

Genes	HR	HR.95L	HR.95H	P
ANPEP	0.822134	0.681012	0.992499	0.041518
AREG	1.154353	1.03309	1.289849	0.011249
COL5A3	0.835201	0.710865	0.981286	0.028548
DKK1	1.161784	1.048932	1.286777	0.004024
FMOD	0.826339	0.715891	0.953827	0.009168
GAS1	0.782505	0.659839	0.927975	0.004814
GPX7	0.818143	0.693531	0.965146	0.017277
PLOD2	1.193821	1.007232	1.414976	0.041046
SFRP1	0.883559	0.799458	0.976508	0.015276
TNFRSF11B	0.638404	0.462455	0.881295	0.00637
VEGFA	1.221469	1.006439	1.482442	0.042874

Abbreviations: HR, hazard ratio; HR.95L, 95% lower confidence interval; HR.95H, 95% upper confidence interval.

### External verification of prognostic potential of this EMT gene signature in OSCC

**FIGURE 2:**
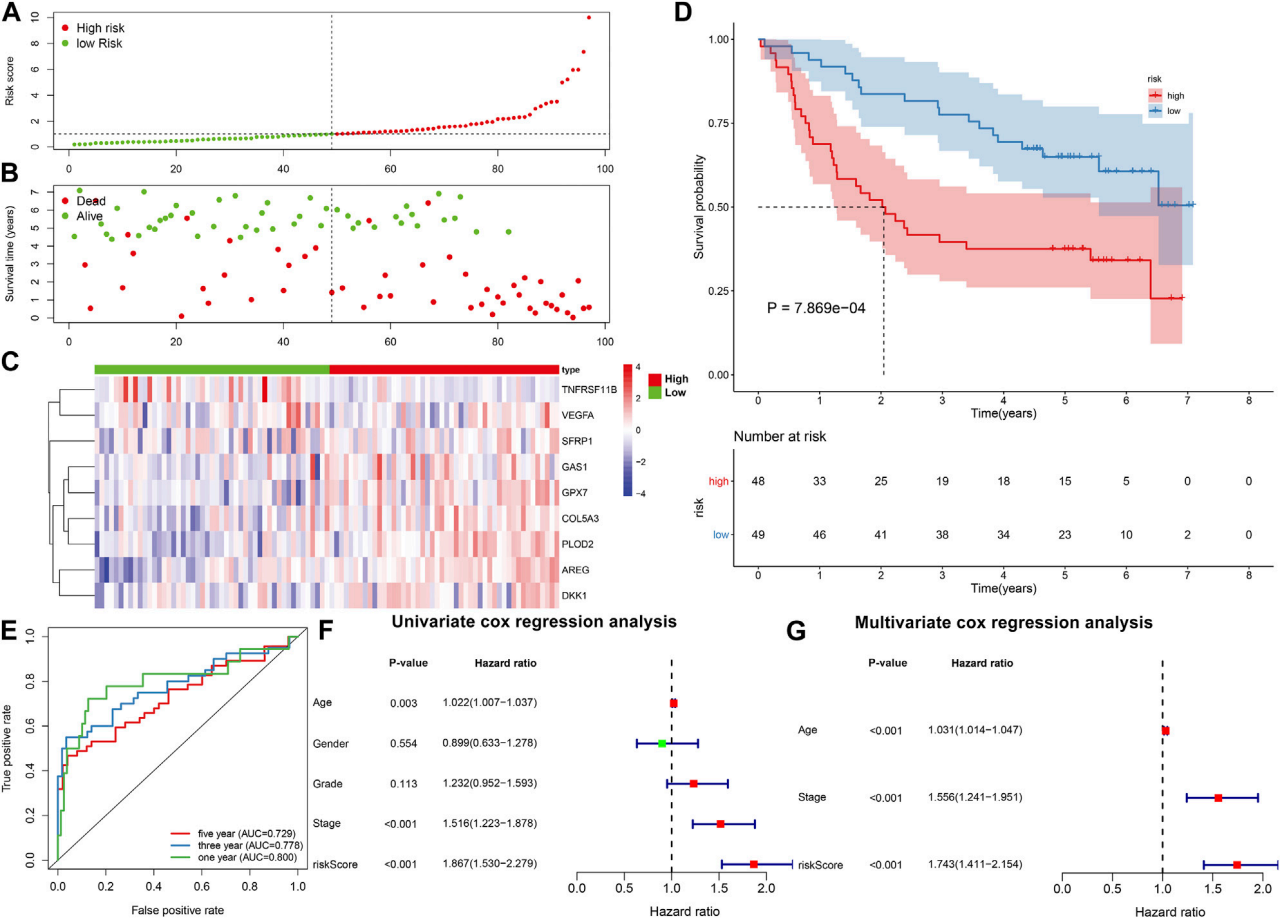
External verification of prognostic value of the EMT gene model in OSCC. **(A)** Distribution of risk scores and identification of high-/low-risk subgroups in the GSE41613 dataset. Vertical dotted line represents the median value of risk score. **(B)** Distribution of survival status in high-/low-risk patients. Red dot represents dead while blue dot represents alive. **(C)** Heat map for expression pattern of genes in this model in high- (red) and low-risk (blue) subgroups. Red indicates upregulation and blue indicates downregulation. **(D)** OS analysis for high-/low-risk patients. *p*-value was calculated with log-rank test. **(E)** ROCs under 1-year, 3-year and 5-year OS based on the risk score. **(F,G)** Univariate and multivariate analyses of the relationships of OSCC prognosis with risk score and other clinical features.

The GSE41613 cohort was employed to externally validate the predictive efficacy of this EMT gene signature in OSCC patients’ prognosis. With the same formula, we calculated the risk scores of OSCC patients. Consistently, we separated OSCC subjects into two subgroups according to the median value of risk score (Figure 2A). Low-risk patients exhibited more optimistic survival status than high-risk individuals (Figure 2B). The expression of the genes in this model was visualized in each OSCC sample (Figure 2C). The survival difference between subgroups was further validated in the GSE41613 dataset. As expected, high-risk patients displayed shorter OS time than low-risk patients (*p* = 7.869e-04; Figure 2D). ROCs were conducted for evaluation of the predictive efficacy of the risk score. In Figure 2E, AUCs under 1-, 3- and 5-year OS were separately 0.800, 0.778 and 0.729, confirming that this risk score could predict OS time of OSCC patients. Univariate analysis revealed that age, stage, risk score displayed distinct associations to OSCC patients’ prognosis in the TCGA dataset (Figure 2F). As confirmed by multivariate analysis, age, stage as well as risk score were independently related to prognosis (Figure 2G).

### Subgroup analysis identifies the sensitivity of the EMT gene signature to predict OSCC prognosis

**FIGURE 3:**
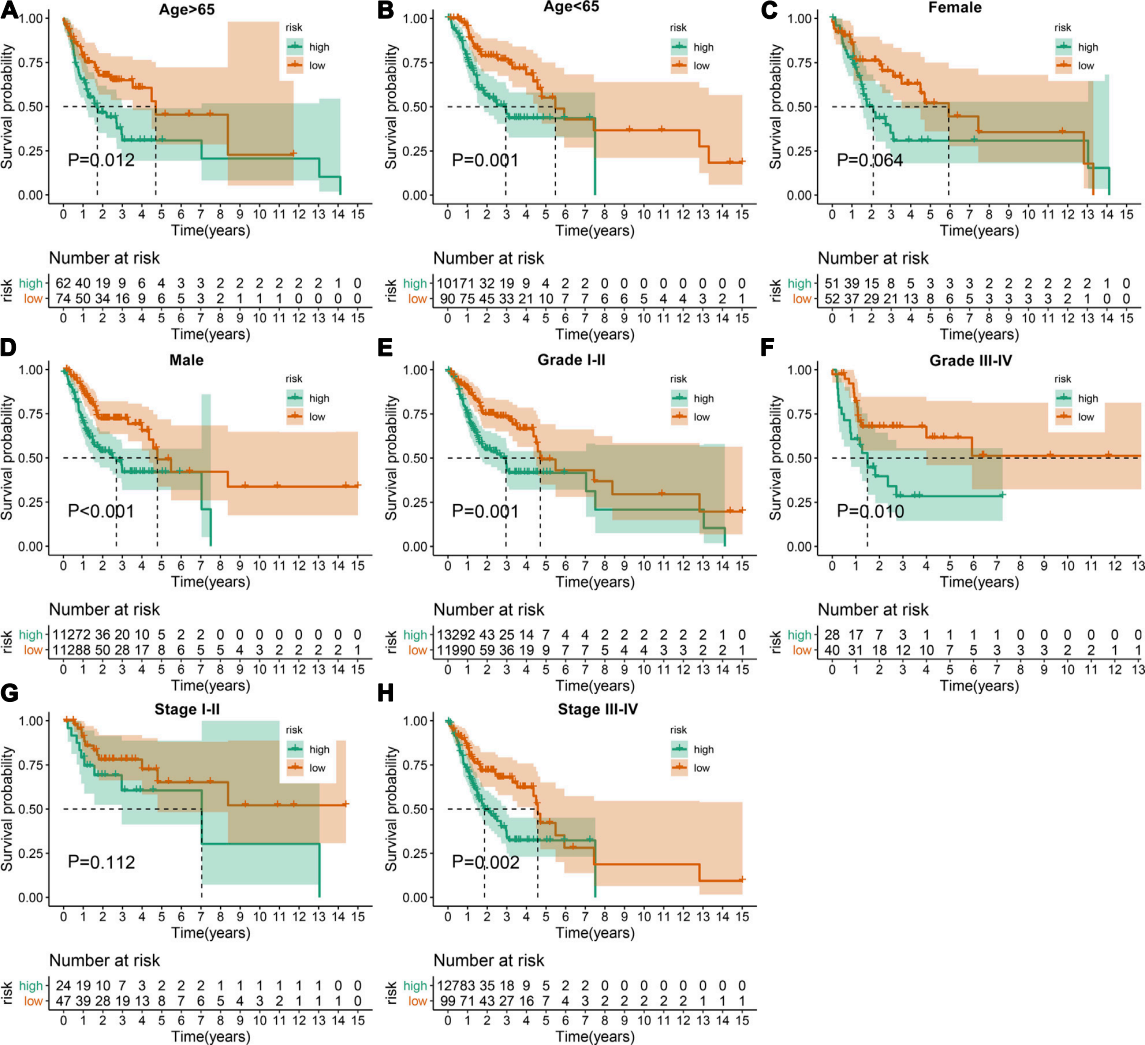
Assessment of the sensitivity of the EMT gene signature to predict OSCC prognosis by subgroup analysis. Kaplan-Meier OS analysis of high- and low-risk patients in **(A)** age >65, **(B)** age <65, **(C)** female, **(D)** male, **(E)** grade I-II, **(F)** grade III-IV, **(G)** stage I-II as well as **(H)** stage III-IV subgroups. *p* values were determined by log-rank test.

The predictive potency of this EMT prognostic model was further evaluated among different subgroups from the discovery cohort. Kaplan-Meier OS analysis showed that high-risk patients were predicted to have worse clinical outcomes compared with low-risk patients in age >65 (*p* = 0.012; Figure 3A), age <65 (*p* = 0.001; Figure 3B), female (*p* = 0.064; Figure 3C), male (*p* < 0.001; Figure 3D), grade I-II (*p* = 0.001; Figure 3E), grade III-IV (*p* = 0.010; Figure 3F), stage I-II (*p* = 0.112; Figure 3G) as well as stage III-IV (*p* = 0.002; Figure 3H) subgroups.

### Association between the EMT gene signature and immune microenvironment in OSCC

**FIGURE 4:**
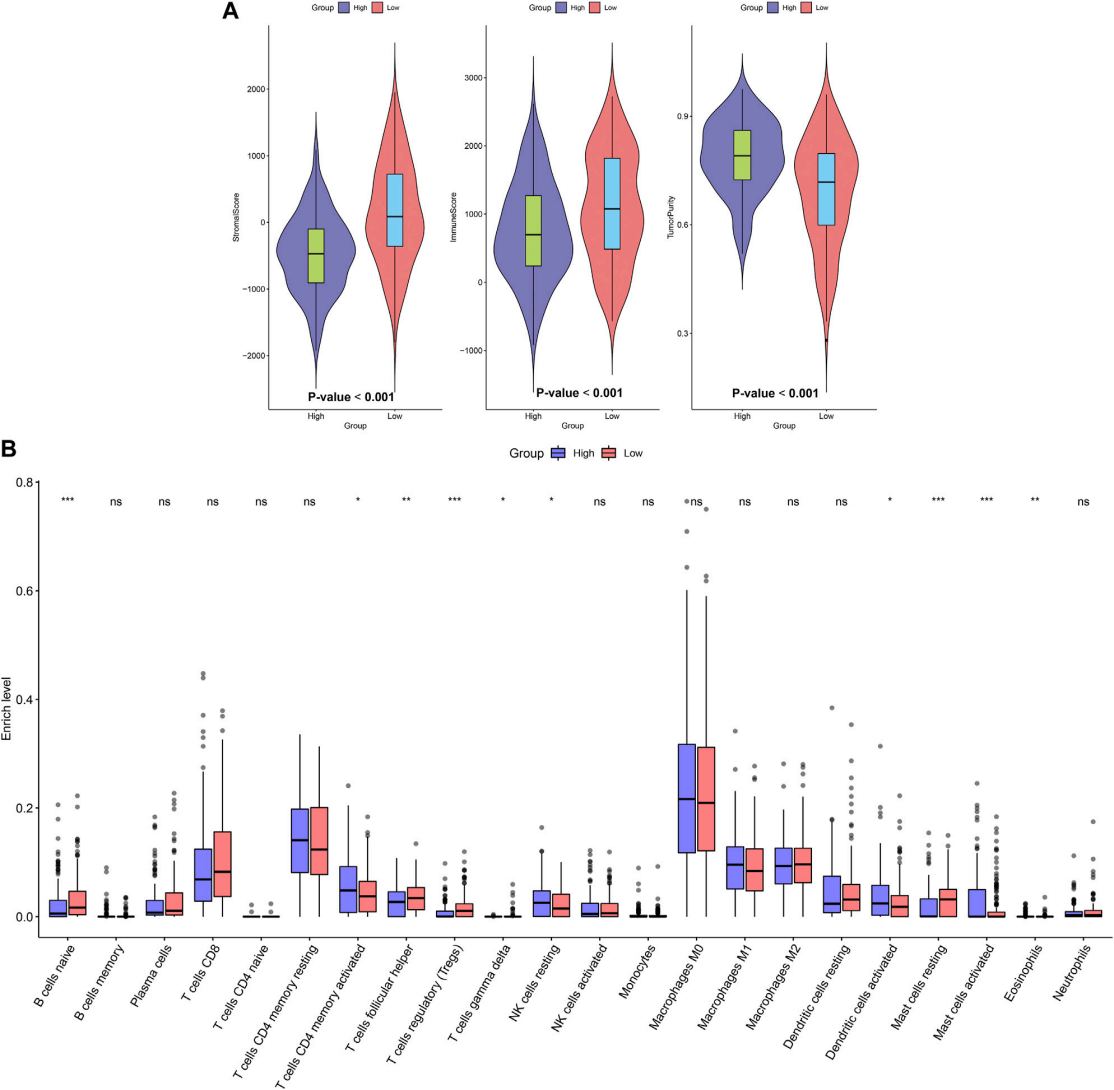
Association between the EMT gene signature and immune microenvironment in OSCC. **(A)** Distributions of stromal score, immune score, and tumor purity in high- and low-risk subgroups. **(B)** Assessment of the infiltration levels of immune cells in high- and low-risk subgroups. *p* values were assessed by Wilcoxon rank-sum test. Ns: not significant; *p < 0.05; **p < 0.01; ***p < 0.001.

ESTIMATE algorithm was employed to assess stromal score, immune score, and tumor purity of OSCC samples from the TCGA dataset. Our data showed that higher stromal (*p* < 0.001) and immune scores (*p* < 0.001) were found in high-risk samples than low-risk samples (Figure 4A). Also, there was lower tumor purity in high-risk samples compared with low-risk samples (*p* < 0.001). The infiltration levels of 22 kinds of immune cells of OSCC specimens were determined by applying CIBERSORT algorithm. There were lowered infiltration levels of naïve B cells (*p* < 0.001), T follicular helper cells (*p* < 0.01), Tregs (*p* < 0.001), T gamma delta cells (*p* < 0.05) and resting mast cells (*p* < 0.001) in high-risk specimens compared with low-risk specimens (Figure 4B). Meanwhile, higher infiltration levels of CD4 memory activated T cells (*p* < 0.05), resting NK cells (*p* < 0.05), activated dendritic cells (*p* < 0.05), activated mast cells (*p* < 0.001) and eosinophils (*p* < 0.01) were examined in high-risk compared with low-risk subgroups.

### Assessment of the EMT gene signature-related signaling pathways and somatic mutation in OSCC

**FIGURE 5:**
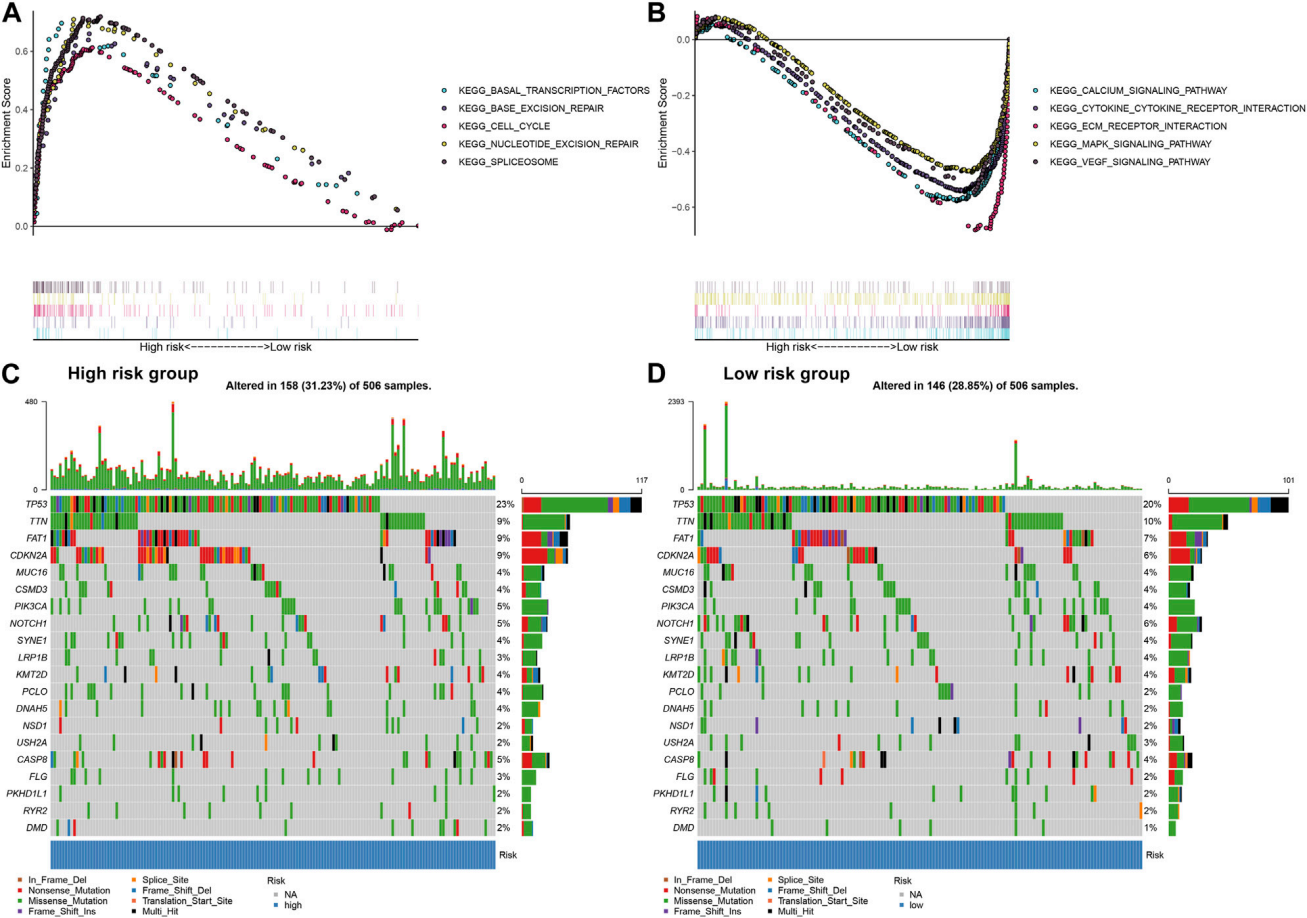
Assessment of the EMT gene signature-related signaling pathways and somatic mutation in OSCC. **(A)** Signaling pathways activated in high-risk subgroup by GSEA. **(B)** Signaling pathways activated in low-risk subgroup. **(C)** The waterfall plots for the first 20 mutated gene signatures in high-risk subgroup. **(D)** The waterfall plots for the first 20 mutated gene signatures in low-risk subgroup.

GSEA was applied to explore signaling pathways associated with the EMT gene signature. As a result, basal transcription factors (NES = 2.04, NOM *p* = 0.002 and FDR = 0.008), base excision repair (NES = 1.82, NOM *p* = 0.006 and FDR = 0.047), cell cycle (NES = 1.96, NOM *p* = 0.006 and FDR = 0.017), nucleotide excision repair (NES = 2.08, NOM *p* = 0.002 and FDR = 0.006) and spliceosome (NES = 2.13, NOM *p* < 0.001 and FDR = 0.004) were distinctly upregulated in high-risk OSCC samples (Figure 5A). Moreover, calcium signaling pathway (NES = −2.21, NOM *p* < 0.001 and FDR <0.001), cytokine-cytokine receptor interaction (NES = −1.99, NOM *p* = 0.002 and FDR = 0.008), ECM receptor interaction (NES = −1.98, NOM *p* = 0.006 and FDR = 0.009), MAPK signaling pathway (NES = −1.94, NOM *p* < 0.001 and FDR = 0.011) and VEGF signaling pathway (NES = −2.18, NOM *p* < 0.001 and FDR <0.001) were activated in low-risk samples (Figure 5B). The somatic mutation was further assessed in high- and low-risk OSCC samples. Our data showed the first 20 mutated genes across OSCC samples. We found that higher frequent genetic mutations occurred in high-risk subgroup (Figure 5C) than low-risk subgroup (Figure 5D), especially TP53, FAT1, and CDKN2A.

### Genes in the EMT gene signature are associated with OSCC prognosis

**FIGURE 6:**
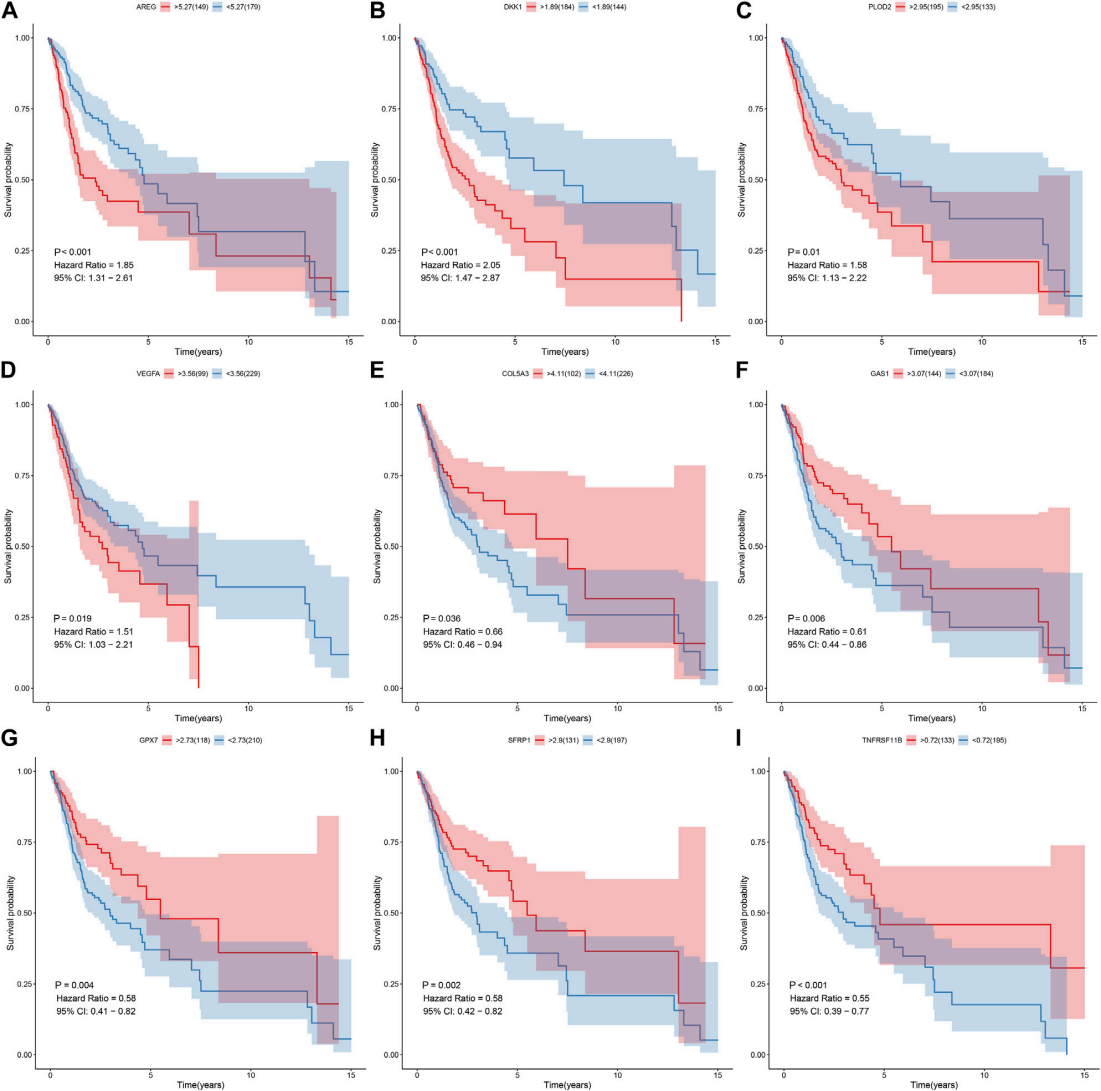
Univariate cox regression analysis for the association between each gene in the EMT gene signature and OSCC prognosis in the TCGA cohort. The survival difference was evaluated between high and low expression of **(A)** AREG; **(B)** DKK1 **(C)** PLOD2; **(D)** VEGFA **(E)** COL5A3; **(F)** GAS1 **(G)** GPX7; **(H)** SFRP1 **(I)** TNFRSF11B subgroups.

**FIGURE 7:**
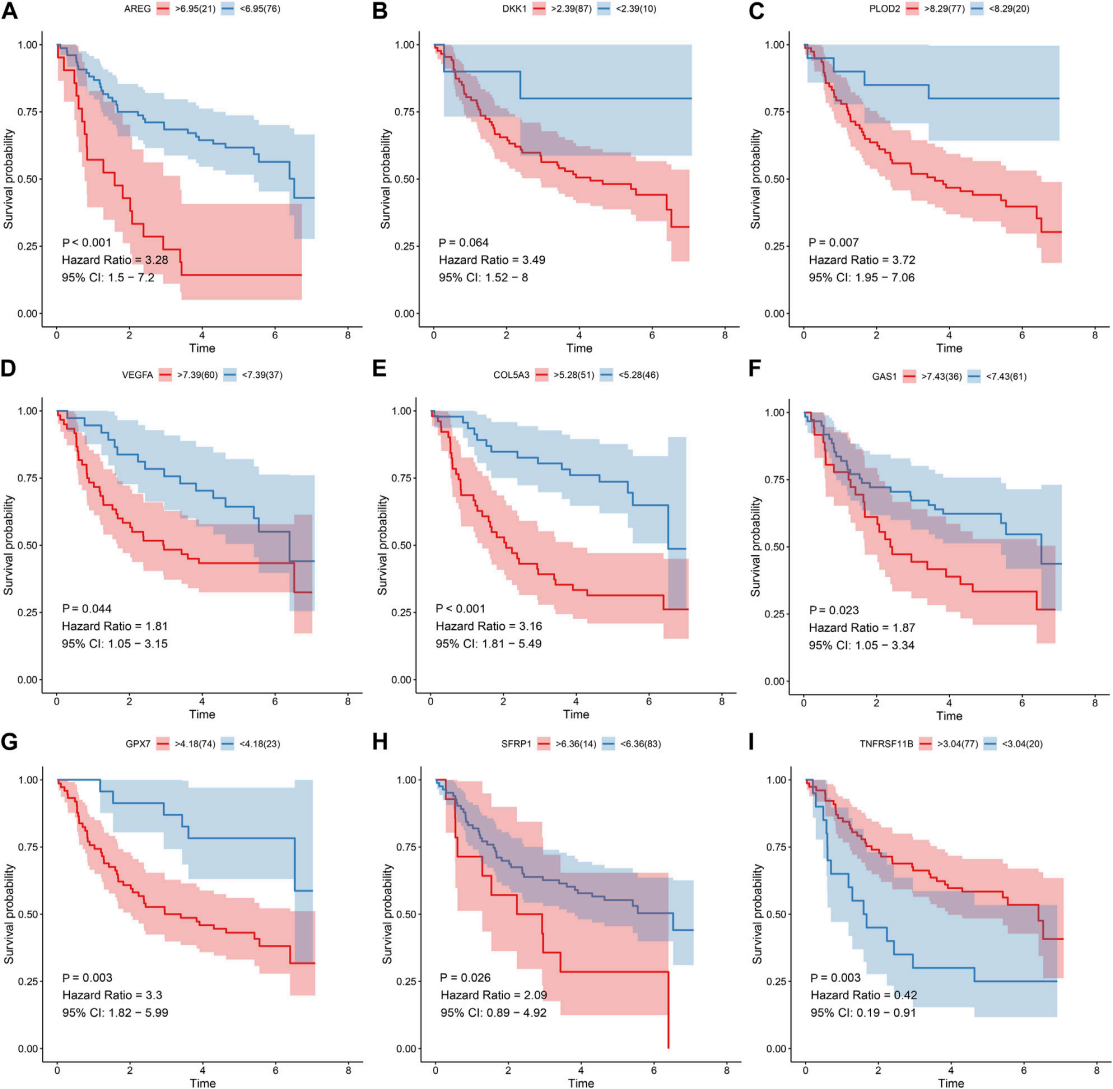
Univariate cox regression analysis for the association between each gene in the EMT gene signature and OSCC prognosis in the GSE41613 cohort. The survival difference was evaluated between high and low expression of **(A)** AREG; **(B)** DKK1 **(C)** PLOD2; **(D)** VEGFA **(E)** COL5A3; **(F)** GAS1 **(G)** GPX7; **(H)** SFRP1 **(I)** TNFRSF11B subgroups.

Prognostic value of each gene in this EMT gene model was evaluated for OSCC samples from the TCGA dataset. Our univariate cox regression analysis demonstrated that high expression of AREG (*p* < 0.001, HR = 1.85, 95%CI: 1.31-2.61; Figure 6A), DKK1 (*p* < 0.001, HR = 2.05, 95%CI: 1.47-2.87; Figure 6B), PLOD2 (*p* = 0.01, HR = 1.58, 95%CI: 1.13-2.22; Figure 6C) and VEGFA (*p* = 0.019, HR = 1.51, 95%CI: 1.03-2.21; Figure 6D) was indicative of poorer prognosis of OSCC patients than their low expression. Furthermore, high expression of COL5A3 (*p* = 0.036, HR = 0.66, 95%CI: 0.46-0.94; Figure 6E), GAS1 (*p* = 0.006, HR = 0.61, 95%CI: 0.44-0.86; Figure 6F), GPX7 (*p* = 0.004, HR = 0.58, 95%CI: 0.41-0.82; Figure 6G), SFRP1 (*p* = 0.002, HR = 0.58, 95%CI: 0.42-0.82; Figure 6H) and TNFRSF11B (*p* < 0.001, HR = 0.55, 95%CI: 0.39-0.77; Figure 6I) displayed significant associations with prolonged survival time compared with their low expression. The prognostic implications of above genes were also confirmed in the GSE41613 cohort (Figures 7A–I).

### Abnormal expression of genes in the EMT gene signature for OSCC

**FIGURE 8:**
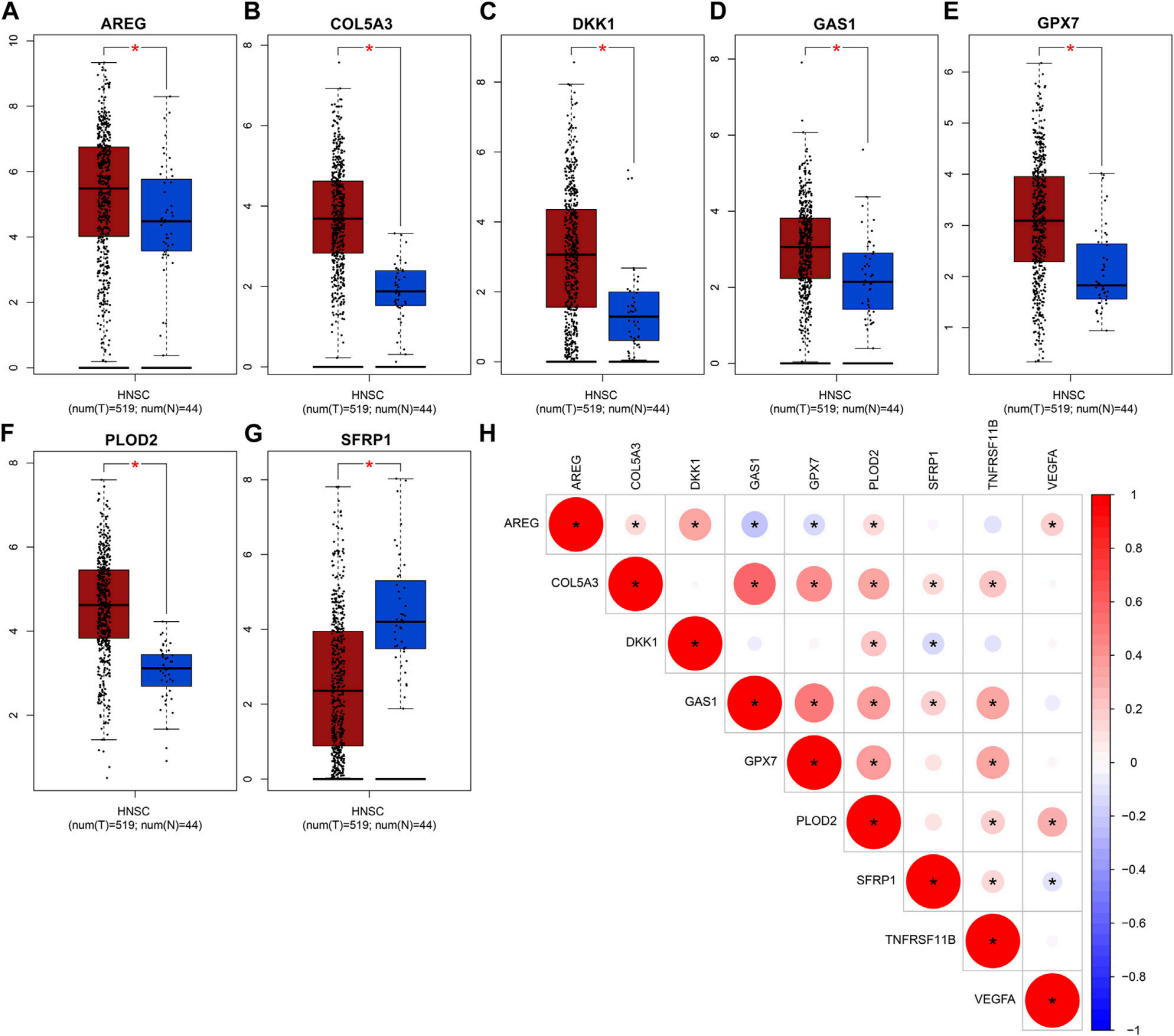
Abnormal expression of genes in the EMT gene signature for OSCC. Box plots for expression of **(A)** AREG, **(B)** COL5A3, **(C)** DKK1, **(D)** GAS1, **(E)** GPX7, **(F)** PLOD2 as well as **(G)** SFRP1 in OSCC and normal tissues. **(H)** Correlation analysis between the genes in the EMT gene signature. Red demonstrates positive correlation as well as blue demonstrates negative correlation. *p < 0.05. The bigger the circle, the stronger the correlation.

The expression of genes in the EMT gene signature was compared between OSCC and normal tissues. Our data showed that AREG (Figure 8A), COL5A3 (Figure 8B), DKK1 (Figure 8C), GAS1 (Figure 8D), GPX7 (Figure 8E) and PLOD2 (Figure 8F) were significantly upregulated in OSCC than normal tissues (all *p* < 0.05). Furthermore, lower SFRP1 expression was found in OSCC compared to normal specimens (*p* < 0.05; Figure 8G). Our correlation analyses demonstrated that COL5A3 exhibited significant correlations to GAS1, GPX7, PLOD2, SFRP1 and TNFRSF11B in OSCC samples (Figure 8H). GAS1 exhibited significant correlations to GPX7, PLOD2, SFRP1 and TNFRSF11B. GPX7 was distinctly associated with PLOD2 and TNFRSF11B. These data indicated that there were distinct correlations between the genes in the EMT gene signature.

### Validation of gene expression in this EMT gene model

**FIGURE 9:**
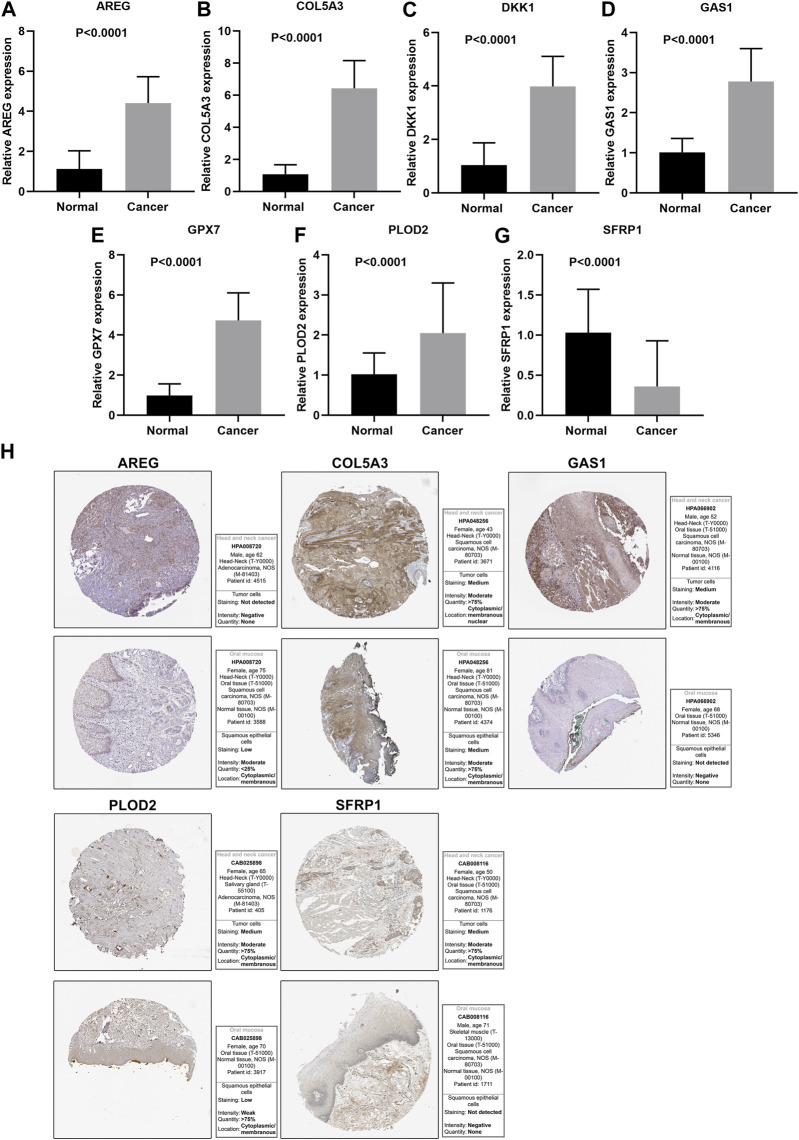
Validation of expression of genes in the EMT gene signature. RT-qPCR for detecting expressions of **(A)** AREG, **(B)** COL5A3, **(C)** DKK1, **(D)** GAS1, **(E)** GPX7, **(F)** PLOD2 and **(G)** SFRP1 in 40 paired OSCC and normal tissue specimens. **(H)** Immunohistochemistry for expression of AREG, COL5A3, GAS1, PLOD2 and SFRP1 in OSCC tissues.

**FIGURE 10:**
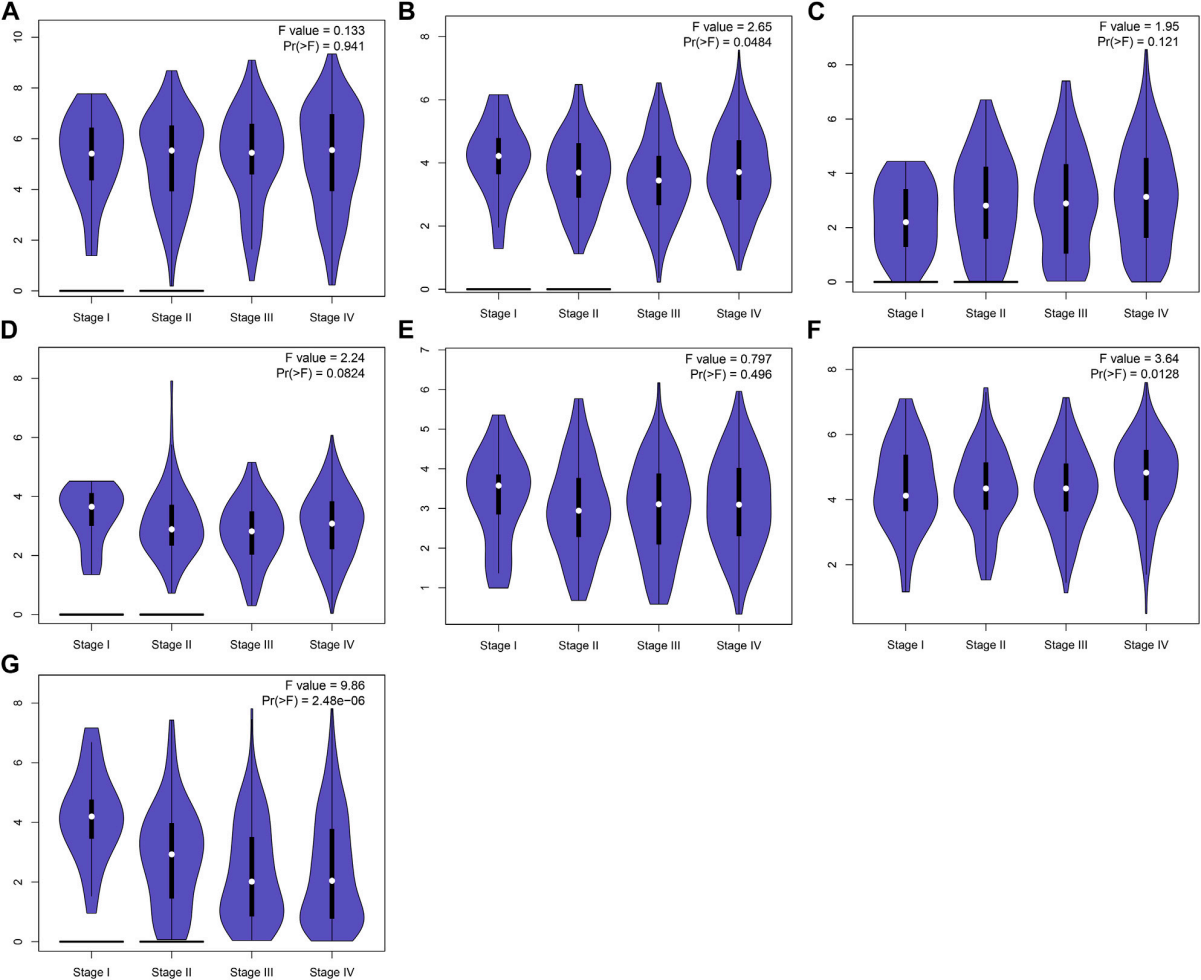
Expression of genes in the EMT gene signature across diverse pathological stages. **(A)** AREG, **(B)** COL5A3, **(C)** DKK1, **(D)** GAS1, **(E)** GPX7, **(F)** PLOD2 and **(G)** SFRP1.

This study further confirmed gene expression in the EMT gene signature between 40 paired OSCC and normal specimens by RT-qPCR. Consistently, our data confirmed that AREG (Figure 9A), COL5A3 (Figure 9B), DKK1 (Figure 9C), GAS1 (Figure 9D), GPX7 (Figure 9E) and PLOD2 (Figure 9F) were distinctly highly expressed in OSCC compared with normal tissues (all *p* < 0.0001). Also, SFRP1 exhibited lower expression in OSCC than normal specimens (*p* < 0.0001; Figure 9G). Abnormal expression of AREG, COL5A3, GAS1, PLOD2 and SFRP1 was also confirmed in OSCC tissues by immunohistochemistry (Figure 9H). We also evaluated the difference in genes from the EMT gene signature across distinct pathological stages, as shown in Figures 10A–G. Among them, COL5A3, PLOD2 and SFRP1 were differentially expressed among pathological stages, indicative of their potential relationships with disease progression.

## Discussion

OSCC represents a progressive malignancy with high heterogeneity (Panarese et al., 2019). Hence, it is of urgency to acquire robust prognostic markers for improving prognosis evaluation and individualized therapy (Zhu et al., 2020). As previous studies, several prognostic signatures have been established for OSCC. For instance, Cao et al. established a 3-mRNA signature (CLEC3B, C6 and CLCN1) in OSCC prognosis (Cao et al., 2019). Hou and colleagues developed an autophagy gene model for speculation of clinical outcomes of OSCC (Hou et al., 2020). Wu and colleagues established an independent transcriptional model according to 5 metabolism pathways concerning OSCC prognosis (Wu et al., 2020). Huang et al. constructed a 7-metabolic gene signature for OSCC (Huang et al., 2021). However, the above gene signatures have not been validated in multiple datasets. Furthermore, so far, no gene signature has been applied in clinical practice. Although many molecular markers and gene signatures have been conducted in OSCC, systematic analyses of expression profiles of EMT genes have not been still performed. In this study, we conducted an EMT gene signature for OSCC prognosis by LASSO method. After external verification, our model robustly and stably predicted patient survival.

The tumor microenvironment contains tumor-associated fibroblasts, immune cells as well as extracellular matrix (Chen et al., 2021). The relationships between tumor microenvironment and tumor cells play key roles in modulating malignant biological behaviors like metastasis and recurrence as well as clinical outcomes of OSCC (Zhou et al., 2020). It has been found that OSCC is highly related to immune infiltration and immune infiltrates are reliable prognostic factors for OSCC (Zhou et al., 2020). For instance, high infiltration of CD103^+^ T and dendritic cells is indicative of prolonged survival outcomes of OSCC (Xiao et al., 2019). Activation of myeloid derived suppressor cells accelerates the malignant progression of OSCC (Pang et al., 2020). Activation of T helper cells in sentinel node indicates unfavorable clinical outcomes in OSCC (Kågedal et al., 2020). Therefore, the variations of immune cell subpopulations in the tumor microenvironments may affect the prognosis of OSCC. Here, our data showed that higher immune or stromal scores were detected in high-than low-risk subgroups. Furthermore, there were lowered infiltration levels of naïve B cells, T follicular helper cells, Tregs, T gamma delta cells and resting mast cells in high-risk than low-risk subgroups. Also, higher infiltration levels of CD4 memory activated T cells, resting NK cells, activated dendritic cells, activated mast cells and eosinophils were examined in high-compared with low-risk subgroups. Thus, this EMT gene signature might be distinctly linked to tumor microenvironment of OSCC.

Our further analysis found that basal transcription factors, base excision repair, cell cycle, nucleotide excision repair as well as spliceosome were activated in high-risk OSCC samples. Consistently, we found that more frequent somatic mutation occurred in high-risk OSCC samples. Calcium signaling pathway, cytokine-cytokine receptor interaction, ECM receptor interaction, MAPK signaling pathway and VEGF signaling pathway were activated in low-risk samples. Previously, calcium-dependent and cell cycle pathways may mediate OSCC progression (Jia et al., 2020). MAPK (Jin et al., 2020) and VEGF pathways (Lien et al., 2020) enhance OSCC progression. These data indicated that the genes in this signature might participate in OSCC pathogenesis by above pathways.

Among the genes in this prognostic signature, AREG, COL5A3, DKK1, GAS1, GPX7 and PLOD2 were distinctly upregulated and SFRP1 was downregulated in OSCC than normal tissues. High expression of AREG, DKK1, PLOD2 and VEGFA was indicative of poorer prognosis of OSCC patients while high expression of COL5A3, GAS1, GPX7, SFRP1 and TNFRSF11B were significantly associated with prolonged survival time. Previously, AREG upregulation has been found in OSCC and it can increase drug resistance against vincristine (Hsieh et al., 2019). Also, AREG expression can independently predict OSCC prognosis (Gao et al., 2016). DKK1 is highly expressed in OSCC and induces proliferation and migration of OSCC cells (Wang et al., 2018). RT-qPCR confirmed the abnormal expression of the genes in OSCC. Combining previous research, the genes in this signature might be potential therapy targets against OSCC. More experiments will be presented for validating their biological functions and clinical implications in OSCC.

## Conclusion

Collectively, based on gene expression profiling, we screened prognosis-related EMT genes and established a 9-EMT gene signature. These data showed that this signature could be utilized to predict clinical outcomes of OSCC subjects, thereby contributing to individual therapy and shedding a novel insight into EMT targeted therapy. Nevertheless, the clinical utility of this signature requires to be verified in a large and prospective OSCC cohort.

## Data Availability

The datasets presented in this study can be found in online repositories. The names of the repository/repositories and accession number(s) can be found in the article/Supplementary Material.

## Abbreviations

AUC, area under the curve; CI, confidence interval; EMT, epithelial-mesenchymal transition; ESTIMATE, Estimation of STromal and Immune cells in MAlignant Tumor tissues using Expression data; GEO, Gene Expression Omnibus; GSEA, Gene Set Enrichment Analysis; HR, hazard ratio; LASSO, least absolute shrinkage and selection operator; NES, normalized enrichment score; OSCC, oral squamous cell carcinoma; OS, overall survival; ROC, receiver operating characteristic; RT-qPCR, real-time quantitative polymerase chain reaction; TCGA, The Cancer Genome Atlas.
